# Postpartum cardiogenic shock due to coronary artery spasm after methylergometrine and sulprostone administration

**DOI:** 10.1007/s12471-023-01805-z

**Published:** 2023-09-08

**Authors:** Pim M. L. Zomer, Maarten P. A. van Teeffelen, Luuk C. Otterspoor, Marlijn J. A. Kamps

**Affiliations:** Department of Intensive Care, Catharina Hospital, Eindhoven, The Netherlands

**Fig. 1:**
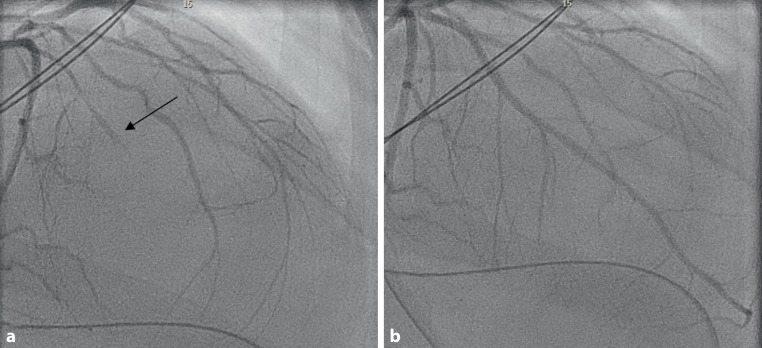
**a** Occlusion of mid-left anterior descending artery (left anterior oblique view 1°, cranial view 30°) before and after intracoronary nitroglycerin. **b** Left anterior descending artery wrapping around the apex (TIMI III flow). (Right anterior oblique view 2°, cranial view 31°)

A 32-year-old pregnant woman was admitted with placental abruption, and a caesarean section was performed. Postpartum haemorrhage ensued, which was treated with oxytocin, methylergometrine and sulprostone. Shortly thereafter she developed ventricular fibrillation and cardiogenic shock. Electrocardiography showed sinus tachycardia with alternating right and left bundle branch block. Transthoracic echocardiography demonstrated a severely impaired left ventricular function with apical and septal akinesia and basal hyperkinesias. Coronary angiography revealed spasm of the left anterior descending coronary artery, which was successfully treated with intracoronary nitroglycerin (Figs. 1a, b). Because of continuing cardiogenic shock, a microaxial flow device was implanted and systemic vasodilators were started. Cardiac contractility recovered rapidly and she was discharged in good clinical condition.

Methylergometrine and sulprostone are frequently used in uncontrolled postpartum bleeding and seldom lead to coronary artery spasm-induced cardiogenic shock [1–4]. In this case, we demonstrated effective treatment with antispasmodic therapy combined with temporary mechanical circulatory support.

### Supplementary Information

CAG before intracoronary nitroglycerin

CAG after intracoronary nitroglycerin
